# Photo-activation of the hydrophobic probe iodonaphthylazide in cells alters membrane protein function leading to cell death

**DOI:** 10.1186/1471-2121-10-21

**Published:** 2009-03-26

**Authors:** Mathias Viard, Himanshu Garg, Robert Blumenthal, Yossef Raviv

**Affiliations:** 1 Nanobiology Program, Center of Cancer Research, National Cancer Institute, Frederick, Maryland, USA; 2 Basic Research Program, SAIC-Frederick Inc, National Cancer Institute, National Institutes of Health, Frederick, Maryland, USA

## Abstract

**Background:**

Photo-activation of the hydrophobic membrane probe 1, 5 iodonaphthylazide (INA) by irradiation with UV light (310–380 nm) results in the covalent modification of transmembrane anchors of membrane proteins. This unique selectivity of INA towards the transmembrane anchor has been exploited to specifically label proteins inserted in membranes. Previously, we have demonstrated that photo-activation of INA in enveloped viruses resulted in the inhibition of viral membrane protein-induced membrane fusion and viral entry into cells. In this study we show that photo-activation of INA in various cell lines, including those over-expressing the multi-drug resistance transporters MRP1 or Pgp, leads to cell death. We analyzed mechanisms of cell killing by INA-UV treatment. The effects of INA-UV treatment on signaling via various cell surface receptors, on the activity of the multi-drug resistance transporter MRP1 and on membrane protein lateral mobility were also investigated.

**Results:**

INA treatment of various cell lines followed by irradiation with UV light (310–380 nm) resulted in loss of cell viability in a dose dependent manner. The mechanism of cell death appeared to be apoptosis as indicated by phosphatidylserine exposure, mitochondrial depolarization and DNA fragmentation. Inhibition by pan-caspase inhibitors and cleavage of caspase specific substrates indicated that at low concentrations of INA apoptosis was caspase dependent. The INA-UV treatment showed similar cell killing efficacy in cells over-expressing MRP1 function as control cells. Efflux of an MRP1 substrate was blocked by INA-UV treatment of the MRP1-overexpressing cells. Although INA-UV treatment resulted in inhibition of calcium mobilization triggered by chemokine receptor signaling, Akt phosphorylation triggered by IGF1 receptor signaling was enhanced. Furthermore, fluorescence recovery after photobleaching experiments indicated that INA-UV treatment resulted in reduced lateral mobility of a seven transmembrane G protein-coupled receptor.

**Conclusion:**

INA is a photo-activable agent that induces apoptosis in various cancer cell lines. It reacts with membrane proteins to alter the normal physiological function resulting in apoptosis. This activity of INA maybe exploited for use as an anti-cancer agent.

## Background

Cells have developed a complex architecture that relies on the compartmentalization of cellular functions within organelles that are bounded by lipid membranes. The plasma membrane constitutes a unique interface between the cytoplasm and extra cellular milieu. While ensuring the physical separation of two very different environments, a constant communication between the cell and its extracellular milieu is established by means of cell surface proteins. Signaling via membrane proteins regulate various cellular functions including cell survival, cell propagation, cell differentiation and cell migration. Therefore membrane proteins are considered prime targets for drugs designed to combat cancer and other diseases. While inhibition of a given cell surface receptor often leads to predictable changes in cells based on the signaling cascade initiated by the receptor, it is unclear how altering cell signaling via a number of receptors would change the cell physiology.

INA is a hydrophobic photo-reactive probe that reacts with transmembrane anchors of membrane proteins upon photo-activation with UV light [1]. INA has been used for the labeling and identification of integral membrane proteins [2-7], in the study of membrane dynamics and fusion [8-10] and for the detection of protein-membrane interactions [10-13]. Recently, we have utilized the transmembrane protein anchor reactivity of INA for inactivation of enveloped viruses [14-17] with preservation of structural integrity for vaccine application. When applied to a variety of enveloped viruses, INA could selectively eliminate functions associated with the hydrophobic domain of the viral envelope required for inducing membrane fusion [16,17]. Based on this premise we hypothesized that treatment of cancer cells with INA-UV would result in inactivation of signaling functions of cellular membrane proteins which in turn would inhibit cell signaling and survival.

With this in mind we conducted the present study to show that INA, a non toxic compound in itself, is highly toxic to a variety of tumor cells including multidrug resistant cells in the presence of UV light. Treatment of cells with INA-UV resulted in inhibition of signal transduction by certain cellular receptors and cell death. The cell death induced by INA-UV showed signs of a classical apoptosis pathway with the involvement of caspases. This study provides evidence that INA can act as a novel and highly active therapeutic agent with a mechanism of action that seems distinct from existing photodynamic therapy compounds.

## Results

### Photo-activation of INA inhibits cell viability

**Figure 1:**
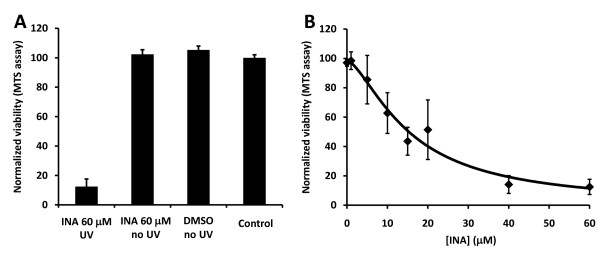
**INA activated by UV prevents MCF7 propagation**. The effect of INA treatment on the propagation of MCF7 cells was measured by a MTS assay. **A**. Cells were treated with 60 μM INA, 1% DMSO or used as is (control). They were then either exposed to UV light or kept in the dark (no UV). MTS measurements were performed 24 h later. **B**. Cells were treated with increasing amounts of INA and irradiated with UV. MTS measurements were performed after 24 h. Data are mean ± S.D of seven measurements.

INA is a hydrophobic compound that partitions in the membrane of cells. Upon irradiation of cells with UV light (310–380 nm) the azido moiety is converted into a highly reactive nitrene that covalently binds membrane proteins. This process leads to the selective inactivation of functions associated with those proteins in the hydrophobic domain of the membrane as has been previously shown for isolated cell membranes [18] and enveloped viruses [14-17]. In this study, we examined the effect of INA-UV on the viability and membrane associated functions of different tumor cell lines. We treated a human breast cancer cell line MCF7 with varying concentrations of INA and irradiated with UV light. As seen in figure 1A, treatment of cells with INA-UV caused significant inhibition of cell viability determined by MTS assay. In the absence of light INA was non toxic to the cells. The effect of INA-UV was dose dependent with IC_50 _of 20.6 μM for MCF7 cells (figure 1B, table 1). The ability of INA-UV treatment to cause cell death was not limited to MCF7 cells. Similar results were obtained with a variety of other cancer cell lines representing cervical cancer, glioma, breast carcinoma, ovarian carcinoma, epidermoid carcinoma and lymphoblastoma (Table 1).

**Table 1:** Cytotoxic activity of INA-UV treatment measured on different cancer cell lines by the MTS assay

**Cell line**	**Human cancer types**	IC_50 _± SE (μM)*
MCF7	Breast adenocarcinoma	20.6 ± 8.8
HeLa	Cervical cancer	11.2 ± 1.7
U251	Glioma	15.4 ± 1.0
SKOV-3	Ovarian carcinoma	17.6 ± 1.0
SKBR-3	Breast carcinoma	7.8 ± 1.5
KB3-1	Epidermoid carcinoma	16.0 ± 0.7
KBV1	Epidermoid carcinoma overexpressing Pgp	12.3 ± 4.4
293	Epithelial cell line from embryonic kidney	8.6 ± 0.9
293/MRP1	Epithelial cell line from embryonic kidney overexpressing MRP1	7.3 ± 1.0
SupT1	T lymphoblastoid	4.9 ± 0.6

* Seven independent measurements were performed and analyzed together. IC_50 _values were computed by fitting a four-parameter nonlinear regression model with *R *statistical software [57]. The standard error (SE) represents the 95% confidence interval.

### Photo-activation of INA induces apoptosis

**Figure 2:**
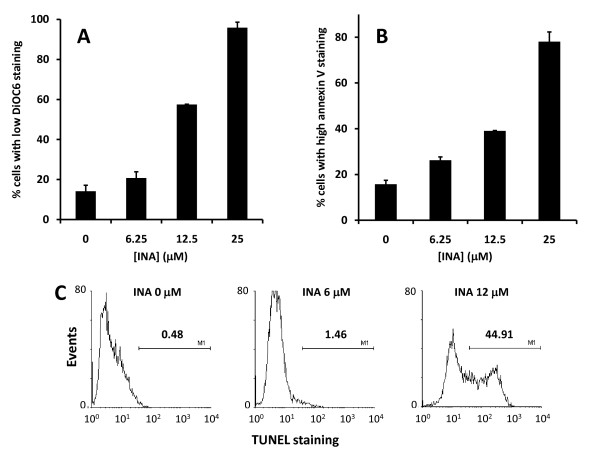
**INA-UV treatment induces apoptosis**. INA-UV treatment of MCF7 cells induces mitochondrial membrane depolarization (DiOC_6 _staining), phosphatidyl serine exposure (Annexin V binding) and DNA fragmentation (TUNEL assay). MCF7 cells treated with increasing amounts of INA and irradiated with UV were stained with DiOC_6 _**(A) **or Annexin V **(B)**. The percentage of the population with low DiOC_6 _staining (depolarized mitochondria) or high Annexin V staining (PS exposure on the cell surface) were determined by FACS analysis and plotted as a function of the INA used for the treatment. Data are mean ± S.D. Representative graphs from two independent experiments are presented. **C**. Histograms corresponding to the TUNEL staining of MCF7 cells treated with different amounts of INA and irradiated with UV.

Next we asked whether the loss of viability seen by INA-UV treatment was due to apoptosis induced in these cells. Apoptosis is characterized by a variety of distinct cellular changes like phosphatidylserine exposure, mitochondrial depolarization and DNA fragmentation. MCF7 cells treated with various concentrations of INA-UV were analyzed for apoptosis markers like mitochondrial depolarization using DiOC_6 _dye (figure 2A) or phosphatidylserine exposure via Annexin V staining (figure 2B). A dose dependent induction of apoptosis was seen in these cells. To further characterize whether the cells were undergoing apoptosis we used the TUNEL assay to detect DNA fragmentation. As seen in figure 2C the cells treated with INA-UV also showed DNA fragmentation confirming the apoptotic mechanism of cell death.

### Apoptosis mediated by INA-UV is caspase dependent

**Figure 3:**
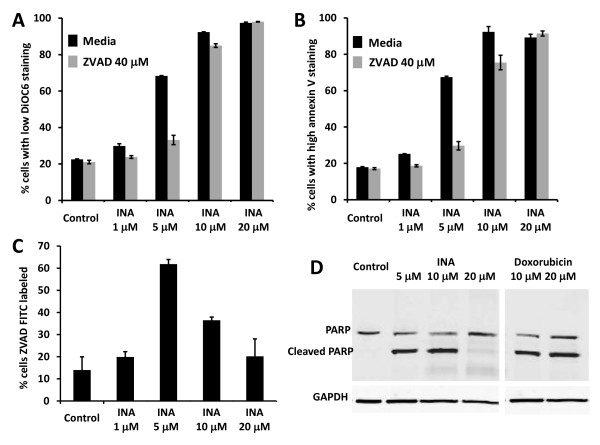
**INA-UV induced apoptosis in SupT1 cells is mediated by caspases**. **A, B**. SupT1 cells, pre-incubated or not with 40 μM ZVADfmk were treated with indicated amounts of INA, irradiated with UV and analyzed 24 h post treatment. **A**. Cells were stained with DiOC_6 _and analyzed by FACS. The percentage of the population presenting low staining (depolarized mitochondria) is presented. **B**. Cells were stained with Annexin V and analyzed by FACS. The percentage of the population presenting high staining (PS exposure) is presented. **C**. Cells were stained with FITC-VAD-FMK and analyzed by FACS. The percentage of the population presenting high staining (caspase activated) is presented. Data are mean ± S.D. Respresentative graphs of two independent experiments are presented. **D**. SupT1 cells were treated with indicated amounts of INA or doxorubicin. 24 hours upon treatment, the cells were lysed and the presence of PARP and/or cleaved PARP was assessed by Western analysis. Loading equivalence was assessed by Western blot analysis of GAPDH.

**Figure 4:**
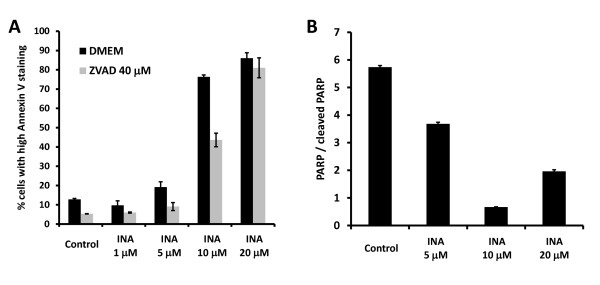
**INA-UV induced apoptosis in Hela cells is also caspase dependent**. **A**. Hela cells, pre-incubated or not with 40 μM ZVADfmk were treated with indicated amounts of INA, irradiated with UV and analyzed 24 h post treatment. Cells were stained with Annexin V and analyzed by FACS. The percentage of the population presenting high staining (PS exposure) is presented. **B**. Hela cells were treated with indicated amounts of INA. 24 hours upon treatment, the cells were lysed and the presence of PARP and/or cleaved PARP was assessed by Western analysis. The ratio of the two as determined by Western analysis is represented.

Caspases are cysteine proteases that are key mediators of apoptosis [19]. The involvement of caspases in the apoptotic pathway can be studied by the inhibition of apoptosis via the pan-caspase inhibitor Z-Val-Ala-Asp-fluoromethylketone (ZVADfmk) and the cleavage of caspase specific substrates like PARP [20,21]. To determine whether apoptosis mediated via INA-UV treatment was caspase dependent, we treated SupT1 cells with ZVADfmk (40 μM) prior to INA-UV treatment. As seen in figure 3A and 3B INA-UV mediated apoptosis at low concentrations was inhibited by ZVADfmk confirming the role of caspases. Interestingly higher concentrations were not inhibited by ZVADfmk suggesting a different mechanism at very high concentrations. In order to monitor caspase activation in SupT1 cells 24 h post treatment with INA-UV, the cells were labeled FITC-VAD-FMK which binds irreversibly with active caspases and analyzed by flow cytometry. As seen in figure 3C, high activation of caspases is observed at 5 μM which correspond to the IC_50 _of INA-UV treatment of SupT1 cells. The activation of caspases was lower at higher doses as determined by FITC-VAD-FMK staining. These findings are supported by the high levels of PARP cleavage seen at low INA concentrations whereas there was little PARP cleavage at higher INA concentrations (figure 3D). PARP cleavage was prevented at all INA concentrations by ZVADfmk indicating its caspase specificity (data not shown). To generalize our conclusion about mechanisms of cell killing, we performed similar experiments with Hela cells. As shown in figure 4, the major caspase involvement in Hela cells takes place at 10 μM INA. At higher INA concentrations ZVADfmk becomes inefficient in preventing apoptosis (figure 4A) and PARP cleavage is decreased (figure 4B). The enhanced caspase activity was observed at 5 μM INA for SupT1 cells and 10 μM INA for Hela cells, which corresponds in each case to the IC_50 _at which INA causes cell death following photo-activation. These studies therefore suggest that in the IC_50 _range of concentrations of INA the apoptosis is mediated by caspases. However, at higher INA concentrations a caspase-independent apoptotic process may be involved.

### INA-UV induced cell killing is not affected by multidrug resistance proteins

**Figure 5:**
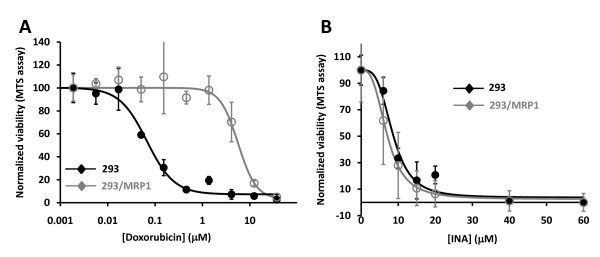
**INA-UV treatment is not affected by the expression of multidrug resistant protein**. The MTS assay was applied to assess the viability of MRP1 overexpressing multidrug resistant 293/MRP1 cell (open circles) and its drug sensitive parent cell line 293 (close circles) subjected to doxorubicin exposure **(A) **or to INA-UV treatment **(B)**. **A**. The cells were exposed to doxorubicin at the indicated concentrations for 72 hours and then subjected to the MTS assay. Data are mean ± S.D. **B**. The cells were subjected to INA-UV treatment and then subjected to the MTS assay 24 hours post treatment. Data are mean ± S.D of seven measurements.

An important problem that arises during chemotherapy is the emergence of drug resistant cells [22]. A major contributor of that phenomenon is an increased efflux of the drugs facilitated by proteins such as the P-glycoprotein (Pgp) or the multidrug resistance protein (MRP) [23-25]. To test whether INA was affected by this phenomenon we used 293/MRP1 cells, a stably transfected 293 cell line that continuously expresses the MRP1 gene at high levels [26]. Indeed, those cell lines exhibited a much lower sensitivity to the commonly used chemotherapeutic agent doxorubicin (figure 5A). However, the IC_50 _of INA-UV cell killing was insensitive to the presence of the multidrug resistance associated gene MRP1 (figure 5B, table 1). Similarly, INA-UV treatment was not significantly affected by Pgp over-expression (table 1).

### INA-UV treatment blocks the MRP1 induced efflux

**Figure 6:**
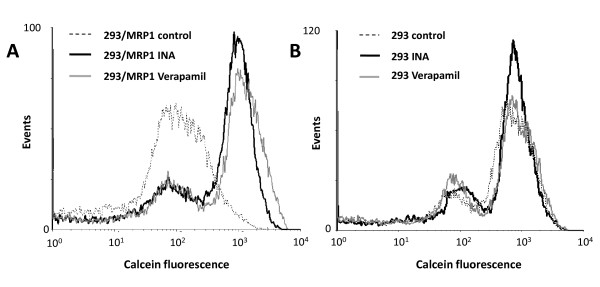
**INA-UV treatment inhibits the calcein efflux of MRP1 expressing cells**. The loading of calcein by 293/MRP1 and 293 cells was assessed by FACS analysis. **A**. Histogram of the calcein fluorescence of 293/MRP1 cells treated or not with 50 μM INA-UV or 40 μM verapamil. **B**. Histogram of the calcein fluorescence of 293 cells treated or not with 50 μM INA-UV or 40 μM verapamil.

MRP1 is a complex transmembrane protein with 17 membrane spanning domains that mediates efflux of various substrates from the cytoplasm of the cell to the extracellular milieu. Calcein is an anionic fluorescent probe that acts as a substrate for MRP1 and determination of its cellular accumulation and efflux allows the investigation of MRP1 activity [27]. As observed in figure 6A, after one hour incubation at 37°C, the 293 cells expressing MRP1 have released a significant portion of the entrapped calcein as evident from the lower fluorescence seen by flow cytometry. This efflux was prevented by incubation of the cells with verapamil (40 μM), an inhibitor of MRP1 function, yielding a labeling efficiency with calcein similar to the one obtained with 293 that do not express MRP1 (figure 6A). Interestingly pre-treatment of the 293/MRP1 cells with INA-UV induced a similar block in MRP1 function as observed with verapamil (figure 6A). On the other hand, in control cells lacking MRP1 neither verapamil nor INA-UV treatment had any significant effect on calcein efflux (figure 6B). This suggests that the increase in calcein labeling in INA-UV treated MRP1 positive cells was in fact due to a specific inhibition of MRP1 function. This is consistent with the inactivation of transmembrane proteins by INA labeling.

### INA-UV affects CXCR4 signaling

**Figure 7:**
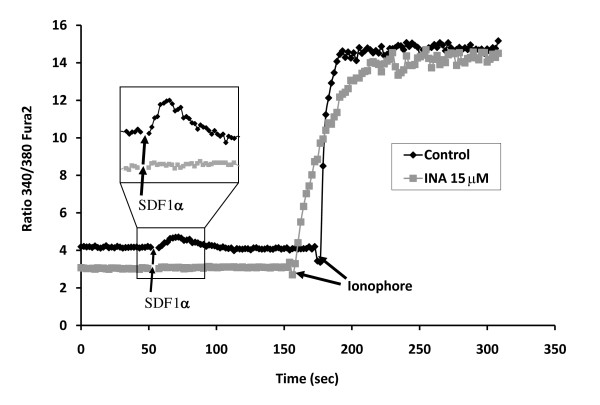
**INA-UV treatment blocks SDF1α induced CXCR4 signaling**. The fluorescence emission of Fura 2 was recorded at 510 nm using simultaneous excitation at 340 and 380 nm. The ratio of the 2 emission signals obtained, 340/380, is plotted over time. SDF1α was added at time 50 sec as indicated and further on the ionophore 4-bromo A-23187 was added at time ~160 sec as indicated. The same experiment was carried on SupT1 cells (dark curve) or SupT1 cells treated with 15 μM INA-UV (grey curve).

CXCR4 is a seven transmembrane G protein-coupled chemokine receptor that is over-expressed in a variety of tumors and involved in tumor metastasis [28]. In cell membranes, blocking the signaling by INA-UV treatment of another G protein-coupled receptor, human chorionic gonadotropin (hCG) has been previously documented [18]. We wished to determine whether INA-UV treatment would alter signaling via CXCR4. Binding of the CXCR4 ligand SDF-1α to its receptor induces a calcium flux in cells, which is monitored by ratio of fluorescent signals at 340 and 380 nm excitation, respectively, and 510 nm emission using the fluorescent calcium indicator dye, Fura 2 pre-loaded into the cells [29,30]. Upon addition of SDF1α we observed calcium flux in the control SupT1 cells but not in cells pretreated with INA-UV (figure 7) suggesting an inhibition of CXCR4 signaling. By contrast, INA-UV treatment had no effect on the action of the calcium ionophore 4-bromo A-23187 that directly mediates calcium flux across membranes. When added to SupT1, in both cases the cytosolic concentration of calcium rose to a similar level as was shown by the increase of the fura 2 ratio signal (Figure 7). Hence the effect of INA-UV was at the level of CXCR4 signaling and not a general effect on intracellular calcium accumulation.

### INA-UV does not inhibit IGF1 signaling

**Figure 8:**
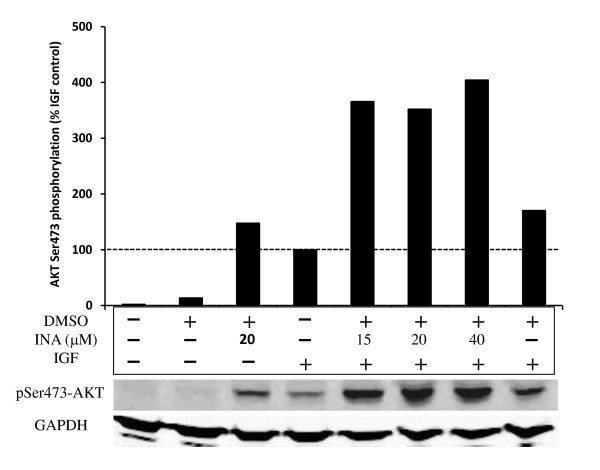
**INA-UV treatment does not inhibit IGF1 signaling**. Akt phosphorylation was assessed by Western detection and quantified. The different samples correspond to MCF7 cells untreated, exposed or not to 1% DMSO, INA at the indicated concentrations and/or IGF1. All the samples exposed to DMSO or INA were irradiated with UV. The Akt phosphorylation signal was normalized to the signal obtained with MCF7 exposed only to IGF1. The Western signal obtained with GAPDH on the same samples is shown for loading control.

Growth factor receptors including EGFR and IGF1R are often overexpressed in tumor cells. Overexpression of IGF1R is associated with increased survival and proliferation of tumor cells. Hence we wished to determine whether INA-UV had an effect on IGF1R signaling and whether it was related to the apoptosis seen in previous experiments. The binding of IGF1 to its receptor activates the PI3kinase pathway leading to Akt phosphorylation at serine 473 [31], which has been reported to be involved in apoptosis inhibition. As can be seen on figure 8, the pretreatment of MCF7 cells with INA-UV leads to an amplification of Akt phosphorylation induced by IGF1 compared to untreated cells. While DMSO partially contributed to this effect, further amplification was observed upon irradiation in the presence of INA. Interestingly we observe that INA-UV treatment alone, in the absence of IGF1, can partially induce Akt phosphorylation in MCF7 cells. This effect requires the reaction to transmembrane proteins with INA, since it is not observed with UV and DMSO treatment alone. These results suggest that INA-UV may in fact activate signaling via IGF1R contrary to the results seen with other receptors. These results underscore the complex physiological outcome of INA interaction with cellular receptors and the diversity in the signal transduction mechanism by different receptors.

### Treatment affects the mobility of membrane proteins

**Figure 9:**
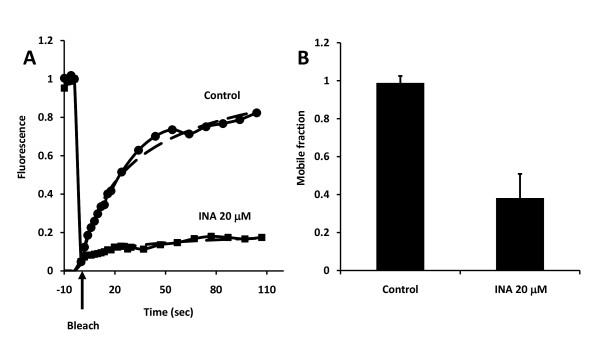
**INA-UV treatment affects the translational diffusion of proteins in the cell membrane**. Fluorescence recovery after photobleaching (FRAP) of CCR5-GFP expressed in Hela cells treated or not with 20 μM INA-UV. **A**. Fluorescence signal recovery as a function of time after photobleaching. **B**. Computed mobile fraction of CCR5-GFP in control cells and cells treated with 20 μM INA-UV. Data are mean ± S.D of seven measurements.

Our efforts to identify the mechanism of action of INA-UV mediated apoptosis induction suggest a direct effect on membrane proteins. Although CXCR4 and MRP1 functions were inhibited by INA-UV treatment, this was not the case with IGF1R function. Photoactivation of INA results in the covalent binding of the probe to membrane proteins in the lipidic bilayer with no particular specificity. This may alter the function of proteins via a variety of mechanisms including the translational mobility of receptors in the plasma membrane. Hence we sought to assess the effects of INA modifications on the mobility of membrane proteins using CCR5-GFP as a reporter. FRAP (fluorescence recovery after photobleaching) is a commonly used method to detect the mobility of fluorescently tagged protein and lipids in cells. We transiently expressed CCR5-GFP in Hela cells and performed FRAP measurements with or without INA-UV treatment. As shown in figure 9 there is a dramatic impact of INA-UV treatment on fluorescence recovery of CCR5-GFP. Curve-fitting recovery after photobleaching shows that INA-UV treatment greatly affects the mobile fraction of CCR5-GFP molecules (figure 9B). INA-UV treatment results in the immobilization of CCR5-GFP while the effect on the mobility of lipids is not significant [17]. These results suggest that INA via direct interaction with transmembrane protein alters the function and mobility of various cellular receptors.

## Discussion

INA is a highly hydrophobic molecule that partitions with high specificity in cellular membranes [32]. This high hydrophobicity along with its photoactivable property makes it a specific probe for transmembrane anchors of membrane proteins. We have exploited this specificity towards transmembrane anchors to inactivate a variety of viral membrane proteins for design of new vaccine candidates [15,17]. In this study we studied the effects of INA-UV treatment on whole cells. Our results indicate that treatment of cell lines with INA by itself was non toxic; however, in the presence of UV light the treatment mediated loss in viability of numerous cancer cells.

The photoactivation of INA is mediated by its azido group that is sensitive to UV irradiation. Upon UV irradiation, a highly reactive nitrene radical is formed that covalently reacts with transmembrane proteins in its vicinity [1]. Such covalent modifications of proteins has been shown to result in the complete loss of infectivity of several enveloped viruses [14-17] and correlates with the loss of membrane fusion function of the viral fusion proteins [16,17]. The transmembrane segments of the fusion proteins of retroviruses [33] and influenza virus [34] are indeed critical for the full fusion process to take place. Although replacement of the transmembrane anchor of influenza virus hemagglutinin with a lipid-anchor obliterated its full fusion capacity, the lipid-anchored hemagglutinin still promoted the hemifusion phenotype leading to the mixing of the outer layers of the viral and target cell membranes [34]. Consistent with this, INA-UV treatment of influenza preserved the hemifusion phenotype confirming the requirement of the transmembrane anchor for full fusion activity and the specific effect of INA on the transmembrane anchor without effecting the function of extracellular domains [17].

Treatment of cells with INA and UV irradiation resulted in complete loss of viability in a variety of cancer cell lines (table 1). This effect was shown to be dependent on the concentration of INA used and strictly required activation by UV light (figure 1). We show here that INA activation induces cell death via characteristics of apoptosis as determined by mitochondrial membrane depolarization, phosphatidyl serine presentation, PARP cleavage and DNA fragmentation in treated cells. At lower doses of INA, apoptosis could be reversed by ZVADfmk, a potent pan caspases inhibitor. Caspase activation was also detected by FITC-VAD-FMK 24 h post INA-UV treatment showing the involvement of caspases in this process and confirming the apoptotic pathway. However, at higher concentrations of INA, caspase activation is decreased and accordingly ZVADfmk becomes ineffective in preventing mitochondrial depolarization and PS presentation suggesting a caspase independent pathway of apoptosis as seen with other treatments like hexaminolevulinate PDT of lymphoma cells [35]. A caspase independent cell death referred to as sub apoptosis has been previously documented [36]. The death inducing stimulus might be such that apoptosis can be achieved through release of apoptosis inducing factors (AIF) from mitochondria without the participation of caspases [37].

As the dosage of INA is increased, more targets within the transmembrane region and possibly other membrane compartments are likely to be reached by the treatment and this can alter the mechanisms inducing the death of the treated cells. If activated INA will covalently react with any protein that is deeply anchored in the lipid bilayer, the consequence of this covalent modification will depend on the function of this portion of the particular protein. Understanding the effect of INA on cellular proteins is important for determining the mechanism of apoptosis induction by INA and its development as an anti cancer agent.

It has been previously reported that INA-UV treatment of cell membranes induces inactivation of gonadotropin receptor by uncoupling of the response of adenylate cyclase to gonadotropins [38]. While binding of chorionic gonadotropin and the luteinizing hormone to gonadotropin receptor were preserved, the binding failed to induce stimulation of the adenylate cyclase pathway. On the other hand, this pathway was shown to be still functional when stimulated directly with NaF. The luteineizing hormone receptor is a member of the G protein-coupled receptor, a family of receptors displaying seven predicted transmembrane helices. CXCR4, another member of this family, has recently been shown to be overexpressed in many cancer cell lines [39,40]. This overexpression is largely due to the hypoxic conditions in the tumor environment [41] and can favor the metastasis of cancer cells through its ligand SDF1α [42-44]. We show here that INA-UV treatment blocks CXCR4 mediated calcium signaling generated by SDF1α stimulation. At the same time the calcium gradient is preserved in the INA-UV treated cells as it is still sensitive to the effect of calcium ionophores indicating that the integrity of the treated cell membranes is not compromised. These data indicate a direct inactivation of CXCR4 receptor signaling by INA-UV treatment.

Similarly, the activity of MRP1 a member of the ABC transporters superfamily that along with Pgp is involved in the drug resistance phenotype in various cancers [45] was also affected by INA-UV treatment. Although no crystal structure is yet available for this protein, ABC transporters are thought to be composed of clusters of predicted transmembrane helices [46]. Overexpression of drug resistance genes makes cells several orders of magnitudes less sensitive to conventional chemotherapeutic agents. The mechanism of action is not clearly understood but the working hypothesis of "hydrophobic vacuum cleaner" has been proposed [7,45] whereby the hydrophobic chemotherapeutic agents while partitioning into the membrane will interact with the transporter within the lipid domain of the bilayer and be pumped outwards. The ability of INA to covalently react with members of ABC transporters has been previously demonstrated [7]. We show here that this interaction leads to the inhibition of MRP1 function and drug efflux. Our data indicate that cell killing induced by irradiation at given concentrations of INA is not affected by the presence of MRP1 or Pgp. Although INA labeling of MRP1 has previously been demonstrated [7], the lack of difference in IC50 for INA killing between MRP1+ and MRP1- cells suggests that either INA is not a substrate for MRP1 or that the Kd for INA binding to MRP1 is much higher than the IC50 for INA to kill cells by irradiation. Therefore irradiation in the presence of INA, and possibly other hydrophobic alkylating agents, appears to be an effective modality of killing of multi-drug resistant cells.

Growth receptor signaling via IGF1R and EGFR has been known to induce cell survival and proliferation in cancer cells via activation of the PI3Kinase and Akt pathway. The tyrosine kinase IGF1R mediated Akt phosphorylation was not affected by INA treatment in cells incubated with IGF1. Interestingly, increased levels of Akt phosphorylation were observed in INA treated cells in the absence of IGF1 stimulation, which was further enhanced upon incubation with IGF1. This suggests that INA treatment may activate IGF1R consistent with reports that a mutation in the transmembrane anchor of IGF1R can result in activation of the receptor [47]. The effects of INA can be interpreted through intramolecular effects via the chemical modification introduced by the covalent addition of a hydrophobic moiety in the transmembrane segment of a protein. However protein-protein and protein-lipid interactions are also likely to be affected by this modification. Proper signaling within cells relies on a dynamic reorganization upon stimuli of the signaling receptors within different domains of the membrane that are thought to assemble and disassemble for the signaling to proceed [48]. We show here by FRAP analysis that INA-UV treatment considerably reduces the mobile fraction of proteins within plasma membranes. Whether this is due to a partial aggregation of membrane proteins and/or a reorganization of domains to accommodate the enhanced hydrophobicity needs to be further studied. The enhanced basal activation of Akt following INA and light treatment might also be a result of receptor immobilization/redistribution. Furthermore, the ability of IGF1 to stimulate the tyrosine kinase IGF1R receptor is considerably amplified by INA treatment. IGF1R has been shown to relocalize in membrane "raft" microdomains in MCF7 cells upon stimulation with IGF1 [49] and activation of Akt also appears to rely on its membrane redistribution [50] in membrane "raft" microdomains [51]. Nevertheless Akt activation induced by INA treatment does not prevent the cells to undergo apoptosis suggesting the mechanism of cell death induced by INA is independent of Akt signaling pathway.

In this report we show the potency of INA as a novel and efficient photoactivable chemotherapeutic agent. The activity of this treatment is strictly dependent on activation by UV light and is mediated by the covalent reaction of INA with membrane embedded domains of proteins. Unlike conventional photodynamic sensitizer that are dependent on reactive oxygen species for activity, reaction of INA with membrane proteins has been shown to be increased under hypoxic conditions [52]. Such hypoxic conditions are common in tumors micro environment and present a major challenge for other photosensitizers. Furthermore, while INA can be directly activated by UV, an equivalent activation can be obtained through energy transfer processes called photosensitization using a variety of chromophores as photosensitizers [6]. The result presented here show that INA is a very potent light activatable therapeutic agent whose targets and mechanism of action are very different from existing PDT agents. Those properties of INA make it a unique candidate for use in photoactivated cancer chemotherapy.

## Conclusion

While INA by itself is innocuous to cells, INA-UV treatment profoundly affects the physiology of various cancer cell lines by inducing apoptosis. The covalent binding of INA to transmembrane domains of proteins alters the signaling capabilities of various cellular receptors in a complex fashion. While G protein-coupled receptors are completely inactivated, the IGF1 receptor remains sensitive to IGF1 stimulation. The translational diffusion of proteins is also profoundly affected by INA-UV treatment. Interestingly, INA is not a substrate for the major multi drug resistance proteins Pgp and MRP1. Furthermore, we show that INA-UV can prevent the efflux capability of MRP1. Overall, the photoactivation of INA in cells results in a dose dependent apoptosis that can be exploited in anti-cancer treatment.

## Methods

### Cells and reagents

The Phospho-Akt (Ser473) and Poly (ADP-ribose) polymerase (PARP, clone 46D11) antibodies were obtained from Cell Signaling (Danvers, MA). The breast adenocarcinoma cell line MCF7 (obtained from the NCI tumor repository) and the T lymphoblastoid cell line SupT1 were propagated in RPMI (Invitrogen, Carlsbad, CA) supplemented with 10% Fetal Bovine Serum (FBS) (Invitrogen, Carlsbad, CA). The cervical cancer cell line HeLa was propagated in Dulbecco's modified Eagle medium (DMEM) (Lonza, Allendale, NJ) supplemented with 5% FBS. The human glioma cell line U251 was a kind gift of Dr Jacek Capala and was propagated in DMEM supplemented with 10% FBS. The human ovarian carcinoma cell line SKOV-3 and the human breast carcinoma cell line SKBR-3 were a kind gift of Dr Jacek Capala and were propagated in McCOY's 5A (ATCC, Manassas, VA) supplemented with 10% FBS. HEK 293 (293) is an epithelial cell line from embryonic human kidney. 293/MRP1 was a kind gift of Dr Suresh Ambudkar. It is a 293 clone that stably expresses multidrug resistant protein (MRP1) and is propagated in DMEM supplemented with 10% FBS and etoposide (5 μM). KB3-1 is a human epidermoid carcinoma and KBV1 is a KB3-1 variant that overexpresses P glycoprotein (Pgp) [53]. All propagation media were supplemented with the antibiotics penicillin-streptomycin (Invitrogen, Carlsbad, CA). Fura 2 and 4-bromo A-23187 were obtained from Invitrogen (Carlsbad, CA). RadioImmunoPrecipitation Assay (RIPA) buffer was obtained from Upstate (Lake Placid, NY). All other reagents were obtained from Sigma (Saint Louis, MO).

### Irradiation procedure

INA was added to the cells from a 100× stock solution in DMSO so that the final concentration desired was always achieved with a 1% final DMSO concentration for all samples. The cells were irradiated with UV light using a 100-W ozone-free mercury arc lamp placed in a lamp house with a collector lens (Olympus) as the light source. Samples were irradiated through a 310-nm cutoff filter placed in front of the lens (to allow transmission of the 313-, 334-, and 365-nm mercury emission bands) and through a water filter (to prevent sample heating) at a distance of 5 cm from the light source. At that point, the light dose was 10 mW/cm^2 ^s. Irradiation times were 2 minutes.

### MRP1 calcein flux measurement

293 cells or 293/MRP1 cells were irradiated with UV in the presence of INA and incubated in medium for 30 minutes at 37°C. In some cases, in order to prevent MRP1 function, the cells were then pretreated with 40 μM verapamil for 30 minutes prior to calcein loading. The cells were then labeled by incubation with 0.25 mM calcein AM for 30 minutes at 37°C. Cells were then washed twice with PBS and the retained intracellular calcein was further determined by fluorescence-activated cell sorting (FACS) analysis on a FACScalibur flow cytometer (BD Bioscience). At least 10,000 events were collected and analyzed using Cell quest software.

### IGF1 mediated signal transduction

Signaling was measured by following the insulin-like growth factor 1 (IGF1) dependent phosphorylation of Akt protein [31]. MCF7 cells were plated in a 12 well plate in RPMI. The medium was replaced with serum free RPMI for overnight incubation. INA was added from 100× stock solution in DMSO for each sample and the samples were irradiated with a UV lamp as described above. IGF1 (R&D systems, Minneapolis, MN) was then added to the medium at a final concentration of 40 ng/ml and the samples were incubated for 10 minutes at 37°C. The cells were then scraped on ice in PBS. The cells were lysed in RIPA buffer supplemented with 2 mM sodium orthovanadate. Proteins from the lysate were separated by electrophoresis and subjected to Western blot analysis for the detection of phosphorylated Akt.

### Western blot analysis

Upon blotting, the membranes were incubated for 1 h in Odyssey blocking buffer (LICOR, Lincoln, NE). The blots were then incubated with the appropriate primary antibody for 2 h at room temperature in Odyssey blocking buffer containing 0.2% Tween-20. The following dilutions were used: 1/1000 for phospho-Akt, 1/4000 for Glyceraldehyde 3-phosphate dehydrogenase (GAPDH) and 1/500 for PARP. Upon four washes of 10 min each with 0.1% Tween-20 in PBS (PBST), the membranes were incubated one hour with anti rabbit 800 and anti mouse 700 (LI-COR, Lincoln, NE) at a dilution of 1/5000 in Odyssey blocking buffer with 0.2% Tween-20, and washed four times for 10 min with PBST. Immunoreactivity was detected by using an Odyssey infrared imaging system (LI-COR, Lincoln, NE).

### CXCR4 chemokine induced signaling

SupT1 cells were treated with 15 μM INA and labeled with 5 μM of Fura 2 for 30 minutes at room temperature. Calcium flux was monitored in a FluoroMax-3 fluorimeter from Horiba Jobin-Yvon (Edison, NJ, USA). The cells were resuspended at 10^6^/ml and the fluorescence emission was recorded at 510 nm using simultaneous excitation at 340 and 380 nm. The calcium flux was triggered by the addition of the chemokine SDF1α (PeproTech, Rocky Hill, NJ) at a final concentration of 35 ng/ml. Complete equilibration of the intracellular and extracellular calcium concentration was achieved by the addition of the ionophore 4-bromo A-23187 at a final concentration of 5 μM.

### Apoptosis

Apoptosis was detected in cells 24 h post treatment using various assays. Mitochondrial depolarization was detected by staining with 10 nM 3,3'-dihexyloxacarbocyanine iodide (DiOC_6_) for 30 min at 37°C followed by flow cytometry. Annexin V-FITC was used to detect PS exposure on cells using ApoAlert kit (BD bioscience). DNA fragmentation was detected by terminal uridine deoxynucleotidyl transferase dUTP nick end labeling (TUNEL) using In situ cell death detection kit (Roche). For PARP cleavage analysis, SupT1 cells were treated with various amounts of INA or doxorubicin. 24 hours upon treatment, the cells were lysed with RIPA buffer for electrophoresis and Western detection. CaspACE™ FITC-VAD-FMK (Promega) was used following manufacturers instruction to detect caspase activation by FACS analysis 24 h post treatment.

### FRAP measurement and analysis

Fluorescence recovery after photobleaching (FRAP) was performed as previously described [54] using a Zeiss LSM 510 (Carl Zeiss, Jena, Germany) confocal laser scanning microscope. HeLa cells were plated on 35 mm glass bottom dishes (MatTek, Ashland, MA) and transfected with CCR5-GFP (a kind gift from Dr Santos-Manes [55]) 24 hours prior to confocal analysis as described previously [54]. INA-UV treatment was performed as described above. The cells were then submitted to FRAP while kept at physiological conditions of 37°C and 5% CO_2 _in a stage incubation system (Incubator S; PeCon GmbH, Erbach, Germany). A 488 nm Ar^+ ^laser line was used for excitation and emission light was collected with a 500–550 bandpass filter. A 40×/1.3 NA oil immersion objective lens was used with a zoom factor of 4. The detector pinhole was opened slightly to acquire an optical section of 2 μm thickness. This allowed more light to be collected for better quantification. Three pre-bleach images were acquired to determine the rate of non-purposeful photobleaching. Photobleaching was performed by increasing the transmission of the laser to 100% for 20–50 iterations to optimize the extent of bleaching. Following photobleaching 8–10 images were acquired at one second intervals and then the acquisition rate was changed to ten second intervals to follow the recovery to completion. A total of 20–40 images were acquired. FRAP analysis was performed using the MIPAV (CIT/NIH, Bethesda, MD) software package using a 1D diffusion FRAP model to retrieve the mobile fraction [56]. Data were automatically corrected with background subtraction, as well as normalization for the non-purposeful photobleaching rate calculated from the whole cell membrane.

### MTS assay

The cells were plated a day before in a 96 wells plate at a density of 2 10^4^/well. The cells were then subjected to UV irradiation in the presence of INA. A day after the treatment, the cells were submitted to a 3-(4,5-dimethylthiazol-2-yl)-5-(3-carboxymethoxyphenyl)-2-(4-sulfophenyl)-2H-tetrazolium, inner salt (MTS) assay (CellTiter 96^® ^AQ_ueous _One, Promega) as per the manufacturer's instructions. For IC_50 _calculation, seven independent measurements were performed and analyzed together. IC_50 _values were computed by fitting a four-parameter nonlinear regression model with *R *statistical software [57]. The standard error (SE) represents the 95% confidence interval.

## Abbreviations

Akt: Akt or protein kinase B; DiOC_6_: 3,3'-dihexyloxacarbocyanine iodide; FACS: Fluorescence-activated cell sorting; FRAP: Fluorescence recovery after photobleaching; GAPDH: Glyceraldehyde 3-phosphate dehydrogenase; IGF1: Insulin-like growth factor 1; IGF1R: Insulin-like growth factor 1 receptor; INA: 1, 5 iodonaphthylazide; MDR or Pgp: P-glycoprotein; MRP1: Multidrug Resistant Protein; MTS: 3-(4,5-dimethylthiazol-2-yl)-5-(3-carboxymethoxyphenyl)-2-(4-sulfophenyl)-2H-tetrazolium, inner salt; PARP: Poly (ADP-ribose) polymerase; PDT: Photodynamic therapy; RIPA: RadioImmunoPrecipitation Assay; SDF1α: stromal cell-derived factor-1; TUNEL: Terminal uridine deoxynucleotidyl transferase dUTP nick end labeling; UV: Ultraviolet rays (310–380 nm).

## Authors' contributions

RB, YR, HG and MV conceived the study. MV and HG designed and performed the experiments. MV drafted the manuscript that was developed with feedback from all the authors. All authors read and approved the final manuscript.

## References

[B1] Viard M, Ablan SD, Zhou M, Veenstra TD, Freed EO, Raviv Y, Blumenthal R (2008). Photoinduced Reactivity of the HIV-1 envelope glycoprotein with a membrane-embedded probe reveals insertion of portions of the HIV-1 Gp41 cytoplasmic tail into the viral membrane. Biochemistry.

[B2] Bercovici T, Gitler C (1978). 5-[125I]Iodonaphthyl azide, a reagent to determine the penetration of proteins into the lipid bilayer of biological membranes. Biochemistry.

[B3] Roess DA, Rahman NA, Munnelly H, Meiklejohn BI, Brady CJ, Barisas BG (1998). Luteinizing hormone receptors are associated with non-receptor plasma membrane proteins on bovine luteal cell membranes. Biochim Biophys Acta.

[B4] Kahane I, Gitler C (1978). Red cell membrane glycophorin labeling from within the lipid bilayer. Science.

[B5] Holowka D, Gitler C, Bercovici T, Metzger H (1981). Reaction of 5-iodonaphthyl-1-nitrene with the IgE receptor on normal and tumour mast cells. Nature.

[B6] Raviv Y, Salomon Y, Gitler C, Bercovici T (1987). Selective labeling of proteins in biological systems by photosensitization of 5-iodonaphthalene-1-azide. Proc Natl Acad Sci USA.

[B7] Raviv Y, Pollard HB, Bruggemann EP, Pastan I, Gottesman MM (1990). Photosensitized labeling of a functional multidrug transporter in living drug-resistant tumor cells. J Biol Chem.

[B8] Raviv Y, Viard M, Bess J, Blumenthal R (2002). Quantitative measurement of fusion of HIV-1 and SIV with cultured cells using photosensitized labeling. Virology.

[B9] Blumenthal R, Pak CC, Raviv Y, Krumbiegel M, Bergelson LD, Morris SJ (1995). Transient domains induced by influenza haemagglutinin during membrane fusion. Mol Membr Biol.

[B10] Pak CC, Puri A, Blumenthal R (1997). Conformational changes and fusion activity of vesicular stomatitis virus glycoprotein: [125I]iodonaphthyl azide photolabeling studies in biological membranes. Biochemistry.

[B11] Gilk SD, Raviv Y, Hu K, Murray JM, Beckers CJ, Ward GE (2006). Identification of PhIL1, a novel cytoskeletal protein of the Toxoplasma gondii pellicle, through photosensitized labeling with 5-[125I]iodonaphthalene-1-azide. Eukaryot Cell.

[B12] Pak CC, Krumbiegel M, Blumenthal R, Raviv Y (1994). Detection of influenza hemagglutinin interaction with biological membranes by photosensitized activation of [125I]iodonaphthylazide. J Biol Chem.

[B13] Merezhinskaya N, Kuijpers GA, Raviv Y (1998). Reversible penetration of alpha-glutathione S-transferase into biological membranes revealed by photosensitized labelling in situ. Biochem J.

[B14] Sharma A, Raviv Y, Puri A, Viard M, Blumenthal R, Maheshwari RK (2007). Complete inactivation of Venezuelan equine encephalitis virus by 1,5-iodonaphthylazide. Biochem Biophys Res Commun.

[B15] Warfield KL, Swenson DL, Olinger GG, Kalina WV, Viard M, Aitichou M (2007). Ebola virus inactivation with preservation of antigenic and structural integrity by a photoinducible alkylating agent. J Infect Dis.

[B16] Raviv Y, Viard M, Bess JW, Chertova E, Blumenthal R (2005). Inactivation of retroviruses with preservation of structural integrity by targeting the hydrophobic domain of the viral envelope. J Virol.

[B17] Raviv Y, Blumenthal R, Tompkins SM, Humberd J, Hogan RJ, Viard M (2008). Hydrophobic Inactivation of Influenza Viruses confers Preservation of Viral Structure with Enhanced Immunogenicity. J Virol.

[B18] Raviv Y, Bercovici T, Gitler C, Salomon Y (1984). Selective photoinduced uncoupling of the response of adenylate cyclase to gonadotropins by 5-iodonaphthyl 1-azide. Biochemistry.

[B19] Cohen GM (1997). Caspases: the executioners of apoptosis. Biochem J.

[B20] Lazebnik YA, Kaufmann SH, Desnoyers S, Poirier GG, Earnshaw WC (1994). Cleavage of poly(ADP-ribose) polymerase by a proteinase with properties like ICE. Nature.

[B21] Slee EA, Zhu H, Chow SC, MacFarlane M, Nicholson DW, Cohen GM (1996). Benzyloxycarbonyl-Val-Ala-Asp (OMe) fluoromethylketone (Z-VAD.FMK) inhibits apoptosis by blocking the processing of CPP32. Biochem J.

[B22] O'Connor R, Clynes M, Dowling P, O'Donovan N, O'Driscoll L (2007). Drug resistance in cancer – searching for mechanisms, markers and therapeutic agents. Expert Opin Drug Metab Toxicol.

[B23] Gottesman MM, Ling V (2006). The molecular basis of multidrug resistance in cancer: the early years of P-glycoprotein research. FEBS Lett.

[B24] O'Connor R (2007). The pharmacology of cancer resistance. Anticancer Res.

[B25] Cole SP, Bhardwaj G, Gerlach JH, Mackie JE, Grant CE, Almquist KC (1992). Overexpression of a transporter gene in a multidrug-resistant human lung cancer cell line. Science.

[B26] Sauna ZE, Peng XH, Nandigama K, Tekle S, Ambudkar SV (2004). The molecular basis of the action of disulfiram as a modulator of the multidrug resistance-linked ATP binding cassette transporters MDR1 (ABCB1) and MRP1 (ABCC1). Mol Pharmacol.

[B27] Feller N, Broxterman HJ, Wahrer DC, Pinedo HM (1995). ATP-dependent efflux of calcein by the multidrug resistance protein (MRP): no inhibition by intracellular glutathione depletion. FEBS Lett.

[B28] Balkwill F (2004). The significance of cancer cell expression of the chemokine receptor CXCR4. Semin Cancer Biol.

[B29] Grynkiewicz G, Poenie M, Tsien RY (1985). A new generation of Ca2+ indicators with greatly improved fluorescence properties. J Biol Chem.

[B30] Ablan S, Rawat SS, Viard M, Wang JM, Puri A, Blumenthal R (2006). The role of cholesterol and sphingolipids in chemokine receptor function and HIV-1 envelope glycoprotein-mediated fusion. Virol J.

[B31] Alessi DR, Andjelkovic M, Caudwell B, Cron P, Morrice N, Cohen P (1996). Mechanism of activation of protein kinase B by insulin and IGF-1. EMBO J.

[B32] Bayley H, Knowles JR (1980). Photogenerated reagents for membranes: selective labeling of intrinsic membrane proteins in the human erythrocyte membrane. Biochemistry.

[B33] Lin X, Derdeyn CA, Blumenthal R, West J, Hunter E (2003). Progressive truncations C terminal to the membrane-spanning domain of simian immunodeficiency virus Env reduce fusogenicity and increase concentration dependence of Env for fusion. J Virol.

[B34] Kemble GW, Danieli T, White JM (1994). Lipid-anchored influenza hemagglutinin promotes hemifusion, not complete fusion. Cell.

[B35] Furre IE, Moller MT, Shahzidi S, Nesland JM, Peng Q (2006). Involvement of both caspase-dependent and -independent pathways in apoptotic induction by hexaminolevulinate-mediated photodynamic therapy in human lymphoma cells. Apoptosis.

[B36] Perfettini JL, Kroemer G (2003). Caspase activation is not death. Nat Immunol.

[B37] Cande C, Vahsen N, Garrido C, Kroemer G (2004). Apoptosis-inducing factor (AIF): caspase-independent after all. Cell Death Differ.

[B38] Raviv Y, Bercovici T, Gitler C, Salomon Y (1984). Selective photoinduced uncoupling of the response of adenylate cyclase to gonadotropins by 5-iodonaphthyl 1-azide. Biochemistry.

[B39] Li S, Huang S, Peng SB (2005). Overexpression of G protein-coupled receptors in cancer cells: involvement in tumor progression. Int J Oncol.

[B40] Zlotnik A (2006). Chemokines and cancer. Int J Cancer.

[B41] Schioppa T, Uranchimeg B, Saccani A, Biswas SK, Doni A, Rapisarda A (2003). Regulation of the chemokine receptor CXCR4 by hypoxia. J Exp Med.

[B42] Koizumi K, Hojo S, Akashi T, Yasumoto K, Saiki I (2007). Chemokine receptors in cancer metastasis and cancer cell-derived chemokines in host immune response. Cancer Sci.

[B43] Ratajczak MZ, Zuba-Surma E, Kucia M, Reca R, Wojakowski W, Ratajczak J (2006). The pleiotropic effects of the SDF-1-CXCR4 axis in organogenesis, regeneration and tumorigenesis. Leukemia.

[B44] Burger JA, Kipps TJ (2006). CXCR4: a key receptor in the crosstalk between tumor cells and their microenvironment. Blood.

[B45] Gottesman MM, Pastan I (1993). Biochemistry of multidrug resistance mediated by the multidrug transporter. Annu Rev Biochem.

[B46] Tusnady GE, Sarkadi B, Simon I, Varadi A (2006). Membrane topology of human ABC proteins. FEBS Lett.

[B47] Takahashi K, Yonezawa K, Nishimoto I (1995). Insulin-like growth factor I receptor activated by a transmembrane mutation. J Biol Chem.

[B48] Simons K, Toomre D (2000). Lipid rafts and signal transduction. Nat Rev Mol Cell Biol.

[B49] Manes S, Mira E, Gomez-Mouton C, Lacalle RA, Keller P, Labrador JP (1999). Membrane raft microdomains mediate front-rear polarity in migrating cells. EMBO J.

[B50] Bellacosa A, Chan TO, Ahmed NN, Datta K, Malstrom S, Stokoe D (1998). Akt activation by growth factors is a multiple-step process: the role of the PH domain. Oncogene.

[B51] Hill MM, Feng J, Hemmings BA (2002). Identification of a plasma membrane Raft-associated PKB Ser473 kinase activity that is distinct from ILK and PDK1. Curr Biol.

[B52] Meiklejohn BI, Rahman NA, Roess DA, Barisas BG (1997). 5-iodonaphthyl-1-azide labeling of plasma membrane proteins adjacent to specific sites via energy transfer. Biochim Biophys Acta.

[B53] Akiyama S, Fojo A, Hanover JA, Pastan I, Gottesman MM (1985). Isolation and genetic characterization of human KB cell lines resistant to multiple drugs. Somat Cell Mol Genet.

[B54] Rawat SS, Zimmerman C, Johnson BT, Cho E, Lockett SJ, Blumenthal R (2008). Restricted lateral mobility of plasma membrane CD4 impairs HIV-1 envelope glycoprotein mediated fusion. Mol Membr Biol.

[B55] Gomez-Mouton C, Lacalle RA, Mira E, Jimenez-Baranda S, Barber DF, Carrera AC (2004). Dynamic redistribution of raft domains as an organizing platform for signaling during cell chemotaxis. J Cell Biol.

[B56] Lippincott-Schwartz J, Snapp E, Kenworthy A (2001). Studying protein dynamics in living cells. Nat Rev Mol Cell Biol.

[B57] DeLean A, Munson PJ, Rodbard D (1978). Simultaneous analysis of families of sigmoidal curves: application to bioassay, radioligand assay, and physiological dose-response curves. Am J Physiol.

